# How a Lady May Become a Doctor

**Published:** 1893-09-16

**Authors:** 


### How a Lady May Become a Doctor.

### THE LONDON SCHOOL OF MEDICINE FOR WOMEN.

It is difficult to-day to realise that only a few years ago the doors of the medical profession in this country were almost completely barred against ladies. The story of the struggle that did not end until the right of women to become medical practitioners had been vindicated reads like an old-world tale of revolt against preposterous disabilities. Yet it was not until 1877 that it was made possible for a woman to obtain a registrable qualification in medicine in this country; and it was only the other day that the British Medical Association rescinded a resolution passed in 1878 excluding women from membership. Nevertheless, so rapid has been the growth of public opinion upon this subject that we now take the lady doctor as a matter of course. Indeed, so many ladies have become doctors since 1877 that one wonders whether the time may not come when it will be necessary to speak of "gentlemen" doctors.

It is no longer possible to argue that women are physically and intellectually incapable of making good medical practitioners, and that it is the duty of examining boards and medical school authorities, for the sake of the public and for the sake of the misguided aspirants themselves, to write above their doors—"No women need apply." The law of the survival of the fittest has been allowed to have its work. If women are unfit to be doctors the result will show it; and the demand for lady doctors will cease. If, on the other hand, there is work for medical women to do—and all the experience that we have had goes to show that there is—how can it be right to place obstacles in the way of those who have the ability and the courage to undertake this work?

It is not necessary to tell over again the story of the battle that was fought in Edinburgh in the early seventies by Miss Sophia Jex-Blake and her comrades. That story has been often told—nowhere better than in Dr. Sophia Jex-Blake's own book. Suffice it to say that when it became evident that it would be useless to pursue the struggle in Edinburgh any longer, it was resolved to come to London and to place the movement on a fresh basis. It was then that the London School of Medicine for Women—the first and still the largest and most important school of its kind in this country—came into existence.

The object which the friends of the movement set before themselves was to establish a school of medicine for women, which the examining bodies would not refuse to recognise. In order to secure this, the plan that commended itself to the promoters was to enlist the services as teachers of men who were already teachers in recognised medical schools in the metropolis. To this the effort of Dr. Anstie, in whose early death a few weeks before the school was opened the movement lost one of its most ardent supporters, of Mrs. Garett Anderson, Miss Jex-Blake, and Mrs. Thorne were directed. A staff of lecturers, which represented much of the best skill and teaching power in the medical schools of London, was secured. The building in Henrietta Street (lately rechristened Handel Street) was taken over and suitably fitted up, and the London School of Medicine for Women was opened on October 14th, 1874, with twenty-three students.

But difficulties had by no means wholly disappeared. It still remained that the lady students should be admitted to the practice of a hospital that contained 150 beds at least, and that school and hospital should be recognised by one of the bodies entitled to confer qualification. Not for three years were these two conditions of the usefulness of the school met, and one may imagine the demand that was made during the interval upon the patience and courage of those who had thrown in their lot with the venture. Help first came from Parliament, which in 1876 passed Mr. Russell Gurney's Bill empowering the British medical examining bodies to examine women. But all the examining bodies make it a condition that candidates whom they receive shall have had a training of which they approve. For a time it seemed doubtful whether the School of Medicine for Women would receive the approval which was needed. Recognition came first from Ireland. The King and Queen's College of Physicians, Dublin, decided to admit students from the School of Medicine for Women to the examination for the degree indicated by the somewhat imposing collocation of letters, L.K.Q.C.P.I. and L.M. Not very long after the difficulty as to a hospital was solved. In the month of June, 1877, the Royal Free Hospital, in Gray's Inn Road, with 160 beds, entered into an arrangement under which lady students were admitted to its practice.

From this time it has been plain sailing with this school and with the medical education of women in this country. A victory of the first order was gained when, in 1878, Convocation of the University of London, by a majority of 241 to 132, approved a new charter admitting women to all degrees of the University. Notwithstanding the well-known stringency of the London examinations, not a few ladies have been successful in taking the degrees in medicine of the University. The example set by the University of London has been gradually followed. The examining bodies which admit women to their examinations are at present seven in number. They are: (1) University of London; (2) the Royal University of Ireland; (3) the Conjoint Colleges of Scotland; (4) the Conjoint Colleges of Ireland; (5) the Society of Apothecaries, London; (6) the University of Glasgow; (7) the University of St. Andrews.

The University of Glasgow insists upon a certain period of study at Queen Margaret's College, which is affiliated with the University. The recent recognition by the University of St. Andrews of the London School for Women, and the resolution to admit ladies to examination for its degrees, is of special importance to those who are anxious to obtain a University degree, but who do not care to enter for the London University examinations, and to whom it is not convenient to put in a period of residence at Glasgow.

The number of students at the London School has shown an increase every year; the number in attendance last session was 143. But the London School no longer occupies the position which it held in 1877 of being the only medical school for women in this country. The lady who has resolved to become a doctor has now a choice of ei[illegible]t schools; of these four are open to women only: The London, the Edinburgh School of Medicine for Women, the Medical College for Women, Edinburgh, and Queen Margaret's College, Glasgow. At the remaining four there are both male and female students; these are the School of Surgery, Dublin, Queen's College, Belfast, Queen's College, Cork, and the School of Medicine, Newcastle.

For several reasons, however, the London School, the parent institution, will always hold the premier place. It was therefore with feelings of peculiar interest that a representative of The Hospital made his way on a recent afternoon to Handel Street to keep an appointment which Mrs. Thorne, the honorary secretary of the school, who helped to found it, and who has been untiring in its interests ever since, had been good enough to make. In the report of the school for 1893 it is stated: "The time will soon come when the rebuilding of the school must be undertaken. The present school buildings are very old, and as the number of students increases it will become more and more necessary to better house the school. The council, however, wish to carry on their work as long as they possibly can in the present houses, as certainly not less than £20,000 will be needed when it becomes absolutely necessary to rebuild." While it cannot be doubted that better buildings will before long become necessary, there is a certain picturesqueness about the school as it is now which it will be impossible to impart to new building. Well removed from noise and bustle, inconspicuous from the street, which is surely an advantage in London, with old-fashioned windows and well-kept lawn guarded by high wall and shaded by leafy trees—where could the true student find more congenial atmosphere and surroundings? But within the school is even more attractive than without. Fresh from visiting the several medical schools in London, one was in a position to make comparison, and be it said at once that the comparison was not to the disadvantage of the ladies. The contribution that woman makes to the comfort and beauty of the home has been made to this medical school. The furnishing and arrangement of the rooms spoke of an artistic taste that is conspicuous for its absence in the majority of medical schools for men. But withal, the uses of class room and laboratory as a means to the end of a thorough training in medicine had never been lost sight of. The school has its library abundantly furnished with text books; and its laboratories as well equipped as could be desired. It has its museum, with a goodly show of specimens, anatomical and pathological, including a series of osteological specimens admirably prepared and mounted by the lady curator of the museum. The dissecting room, which is at the top of the building, is all that a dissecting room should be, and it is more comfortable than most. Altogether, as one was courteously shown over the building by Mrs. Thorne, one could not help feeling that fortunate are the ladies who are enabled to pursue their studies in such a favourable environment.

The list of lecturers at the school still bears the names of a number of distinguished men who are also teachers at other schools; but as vacancies have taken place they have been filled up by ladies of approved professional and teaching skill. Thus Mrs. Garett Anderson, M.D., who has been Dean of the school since 1883, shares with Dr. Donkin the work of teaching practice of medicine. Mrs. Scharlieb, M.D., is lecturer on midwifery, and Miss McCall, M.D., has charge of the class of practical midwifery. Miss Lucy Boole has been very successful as a teacher of chemistry. Among other ladies on the staff are Mrs. Dowson, L.R.C.P., who shares the work of the class of forensic medicine with Dr. Dupré, of Westminster Hospital; Miss M. F. Ewart, Elementary biology and physics; Mrs. Flemming, M.D., and Miss Bale, L.R.C.P., demonstrators of anatomy; and Mrs. Clarke Keer, elementary materia medica and pharmacy.

Students at the School of Medicine for Women receive clinical instruction at the Royal Free Hospital. The governing bodies are quite distinct, with the result that the staffs of school and hospital are for the most part different, although one or two gentlemen hold posts at both. School and hospital are, however, on the best of terms. Every provision has been made at the hospital for imparting clinical instruction, and special attention is very properly given to gynæcology.

The fee for the ordinary curriculum of study at school and hospital is £125 if paid in one sum, or £135 if paid in instalments.

Although it is not twenty years since the London School of Medicine for Women came into existence, the number of ladies who have had their training within its walls, and who are now in the practice of their profession at home and abroad is large. But the best object lesson as to the work which the school has done is provided in the new Hospital for Women, 144, Euston Road. With the exception of a few of the consulting physicians and surgeons, the staff of this hospital is composed entirely of ladies. When a body of ladies can be found to undertake a responsibility such as this, and to discharge it with credit to themselves, and with the best of results to the patients who come under their care, who will say that there is not room in the medical profession for women who unite to intellectual aptitude the enthusiasm for lessening the sufferings of humanity without which no man nor woman can become a successful doctor in the true and high sense?

